# MiMeDB: the Human Microbial Metabolome Database

**DOI:** 10.1093/nar/gkac868

**Published:** 2022-10-10

**Authors:** David S Wishart, Eponine Oler, Harrison Peters, AnChi Guo, Sagan Girod, Scott Han, Sukanta Saha, Vicki W Lui, Marcia LeVatte, Vasuk Gautam, Rima Kaddurah-Daouk, Naama Karu

**Affiliations:** Department of Biological Sciences, University of Alberta, Edmonton, AB T6G 2E9, Canada; Department of Computing Science, University of Alberta, Edmonton, AB T6G 2E8, Canada; Department of Laboratory Medicine and Pathology, University of Alberta, Edmonton T6G 2B7, AB, Canada; Faculty of Pharmacy and Pharmaceutical Sciences, University of Alberta, Edmonton, AB T6G 2H7, Canada; Department of Biological Sciences, University of Alberta, Edmonton, AB T6G 2E9, Canada; Department of Biological Sciences, University of Alberta, Edmonton, AB T6G 2E9, Canada; Department of Biological Sciences, University of Alberta, Edmonton, AB T6G 2E9, Canada; Department of Biological Sciences, University of Alberta, Edmonton, AB T6G 2E9, Canada; Department of Biological Sciences, University of Alberta, Edmonton, AB T6G 2E9, Canada; Department of Biological Sciences, University of Alberta, Edmonton, AB T6G 2E9, Canada; Department of Computing Science, University of Alberta, Edmonton, AB T6G 2E8, Canada; Department of Biological Sciences, University of Alberta, Edmonton, AB T6G 2E9, Canada; Department of Biological Sciences, University of Alberta, Edmonton, AB T6G 2E9, Canada; Department of Biological Sciences, University of Alberta, Edmonton, AB T6G 2E9, Canada; Department of Medicine, Duke University School of Medicine, Durham, NC 27710, USA; Leiden Academic Centre for Drug Research, Leiden University, Leiden 2333 CC, The Netherlands

## Abstract

The Human Microbial Metabolome Database (MiMeDB) (https://mimedb.org) is a comprehensive, multi-omic, microbiome resource that connects: (i) microbes to microbial genomes; (ii) microbial genomes to microbial metabolites; (iii) microbial metabolites to the human exposome and (iv) all of these ‘omes’ to human health. MiMeDB was established to consolidate the growing body of data connecting the human microbiome and the chemicals it produces to both health and disease. MiMeDB contains detailed taxonomic, microbiological and body-site location data on most known human microbes (bacteria and fungi). This microbial data is linked to extensive genomic and proteomic sequence data that is closely coupled to colourful interactive chromosomal maps. The database also houses detailed information about all the known metabolites generated by these microbes, their structural, chemical and spectral properties, the reactions and enzymes responsible for these metabolites and the primary exposome sources (food, drug, cosmetic, pollutant, etc.) that ultimately lead to the observed microbial metabolites in humans. Additional, extensively referenced data about the known or presumptive health effects, measured biosample concentrations and human protein targets for these compounds is provided. All of this information is housed in richly annotated, highly interactive, visually pleasing database that has been designed to be easy to search, easy to browse and easy to navigate. Currently MiMeDB contains data on 626 health effects or bioactivities, 1904 microbes, 3112 references, 22 054 reactions, 24 254 metabolites or exposure chemicals, 648 861 MS and NMR spectra, 6.4 million genes and 7.6 billion DNA bases. We believe that MiMeDB represents the kind of integrated, multi-omic or systems biology database that is needed to enable comprehensive multi-omic integration.

## INTRODUCTION

The launch of the human microbiome project in 2006 (1) combined with continuing advances in DNA sequencing, has led to an explosion in interest in characterizing human-associated microbes. For instance, in 2021 alone there were >13 000 papers appearing in PubMed with the terms ‘microbiome’ and ‘human’. This work has revealed how important this previously forgotten part of the human body is, especially with regard to human health and disease. Over the past decade, many significant associations have been found between human gut microflora and gastrointestinal disorders (2), obesity (3), mood (4) and immunity (5). While most human microbiome studies continue to focus on connecting health outcomes to measures of microbial taxonomy (such as microbe types or measures of microbial diversity), there is increasing awareness that it's not the microbes themselves that lead to specific health effects, it's the chemicals they produce.

Indeed, many microbially produced chemicals have long been known to play important roles in human health and disease with some, such as butyric acid, that offer protection against inflammation and cancer (6) and others, such as indoxyl sulfate, promoting inflammation and causing damage to the kidneys, heart and brain (7–11). However, with more focus on combining metagenomics with metabolomics, the list of these microbially derived chemicals and their physiological effects is growing almost weekly (12,13). This work is also revealing that the abundance and composition of these microbial metabolites can be profoundly affected by both diet and chemical or environmental exposures (7,12,13). As a result, the study of the human microbiome has evolved from an almost singular genomic pursuit to a much more holistic, multi-omic enterprise that requires the linking of the microbial (meta)genome, proteome and metabolome to the human exposome—and finally to human health.

Multi-omic studies not only require multi-omic technologies, they also require multi-omic databases and multi-omic informatic (bioinformatic and cheminformatic) tools. Our own work in human microbiome studies (14) revealed that there was a shortage of these kinds of multi-omic databases, especially for integrated microbiome studies. It was also apparent that much of the information needed was scattered in many different journals, books and specialized electronic databases. For instance, general microbial metabolism data is typically contained in metabolism databases such as the Kyoto Encyclopedia of Genes and Genomes (KEGG) (15), BioCyc (16) and the Virtual Metabolic Human (VMH) (17). General microbial genome and/or proteome data is housed in GenBank (18) and UniProt (19), while bacterial chromosome maps are located in BacMap (20). Human exposome and diet data is found in databases such as FooDB (21) or Exposome Explorer (22), while health effect data and microbial metabolite descriptions are available in the Human Metabolome Database (HMDB) (23) or the *E. coli* Metabolome Database (ECMDB) (24). None of these resources are truly connected or integrated and none of them are explicitly focused on the human microbiome.

It was because of these data deficits that we decided to create MiMeDB—the Human Microbial Metabolome Database. MiMeDB is designed to be a fully integrated multi-omic database that links the human microbial (meta)genome, proteome and metabolome to the human exposome—and human health. Our goal in creating MiMeDB was to create a resource that researchers with genomic, proteomic, metabolomic or exposomic data could use to query and interactively explore, visualize and interpret their data with other known multi-omic data on the human microbiome. In developing MiMeDB, we attempted to bring many of the best features and some of the higher-value content from specialized databases such as VMH, HMDB, KEGG, GenBank, UniProt, BacMap, FooDB and HMDB, together into a single resource. These data have then been reformatted and integrated into a web-friendly database that allows users to perform sophisticated queries (sequence, structure, spectral or text) and interactive visualizations (genome views, network views, spectral views, pathway views or structural views). A more detailed description of MiMeDB’s content, its layout and design, as well as additional details about how it was constructed and how it is maintained, are provided in the following pages.

## MiMeDB CONTENT AND BACKEND STRUCTURE

In simple terms, MiMeDB contains data about human microbes, their associated metabolites and the effects these metabolites have on humans. From the microbial side, MiMeDB contains molecular (genomic, proteomic), phenotypic (host or site preference data, gram stain, oxygen requirements, trophism) and taxonomic data about those microbes (including bacteria and fungi) known to reside within or on the human body. From the metabolite side, MiMeDB contains compound descriptions, chemical nomenclature, chemical source/origin, reaction data (with associated enzyme data) as well as physico-chemical and spectroscopic data about the endogenous and exogenous chemicals used, produced or co-metabolized by human microbes. From the effect side, MiMeDB contains data on known or suspected human health effects, biospecimen concentrations, human protein targets and the corresponding bioactivities of the compounds produced or co-produced by human microbes.

In terms of its microbiome content, MiMeDB contains data on 1904 microbes including 1896 bacterial species (1842 eubacteria and 54 archebacteria) as well as eight common fungal species that can be found on or in the human body. Complete taxonomic and genomic sequence data is available for all microbes in MiMeDB. Full genome maps with detailed gene, protein, prophage/provirus, rRNA and tRNA annotation data are also available for all microbes in MiMeDB. In addition, host or body site preference information, gram stain properties, oxygen requirements and trophism data is available for ∼90% of the bacterial species.

In terms of its metabolome content, MiMeDB contains data on 24 254 metabolites or chemicals covering >72 different chemical classes. These metabolites include 1808 microbial-only compounds, 14 210 human-microbe co-metabolites, 8236 human-only metabolites and 1524 exogenous (drug, food, cosmetic, pollutant) compounds. More than 600 different food sources are provided in MiMeDB. Here we define a microbial-only metabolite as a chemical that can only be produced by microbial enzymes or microbially-mediated reactions. To qualify as such, there must be no evidence that the metabolite has an origin other than via a microbe-specific enzyme, process or pathway. A microbial-host co-metabolite is a chemical that arises from a combination of human and microbial metabolism as determined by its reaction sequence, known biochemical pathway(s) or biochemical processes. In other words, the chemical must arise from at least one microbial-specific reaction with an associated microbial enzyme or process and at least one human-specific reaction with a specific human enzyme. A human-only metabolite is a chemical that can only be synthesized or metabolized directly by known human enzymes or human-specific processes and for which no evidence of microbial enzymes or microbial reactions exists. Exogenous compounds are xenobiotic compounds that cannot be endogenously synthesized by microbes or humans and typically include plant-derived food chemicals, drugs, cosmetics or other synthetic compounds.

All metabolites or chemicals in MiMeDB contain ∼90 data fields covering structural data, detailed descriptions, nomenclature data, chemical taxonomy data, ontological data, physico-chemical data, spectral data, biological properties, external links and references. A total of 79% of the compounds in MiMeDB also contain reaction data showing the substrates, products and enzymes responsible for the reactions. MiMeDB also houses experimentally collected and predicted metabolite spectra. This includes 45 743 EI-MS spectra, 120 079 MS/MS spectra (at 10, 20 and 40 eV), 161 785 retention index values, 39 401 collisional cross section (CCS) values, 241 701 ^1^H NMR spectra and 241 338 ^13^C NMR spectra (covering up to 10 different NMR spectrometer frequencies) for almost all metabolites. The methods used to collect the experimental spectra and to perform the spectral predictions are described elsewhere (25,26).

With regard to MiMeDB’s health or health effect content, the database contains information on 626 human diseases, conditions or suspected conditions that arise from abnormal microbial metabolites or abnormal co-metabolite concentrations. A total of 26 172 normal and abnormal biospecimen concentrations (for 18 different biospecimen types) are available for 3066 metabolites. Bioactivity data (corresponding to 489 different bioactivity types) is also available for 4238 metabolites. This bioactivity data is associated with 104 human (protein) targets. Pathway data is available for 19 389 metabolites. A more detailed listing of MiMeDB’s other content and statistics is available on the MiMeDB website via the **About** menu tab under the ‘Statistics’ section.

While the general content of MiMeDB can be grouped into three broad classes (microbes, metabolites and effects), MiMeDB is actually divided into six smaller categories for more facile browsing, searching and viewing. These six categories include the: (a) ‘Metabolite’ category, (b) ‘Microbial Sources’ category, (c) ‘Host Biospecimen & Location’ category, (d) ‘Health Effect & Bioactivity’ category, (e) ‘Exposure Sources’ category and (f) ‘Metabolic Reactions’ category. The ‘Metabolite’ category functions as a central hub category which is logically and literally connected to the five other MiMeDB categories (Microbial Sources, Exposure Sources, Biospecimen & Location, Health Effect & Bioactivity as well as Metabolic Reactions). The ‘Metabolite’ category contains 11 major data fields, covering information on metabolite identification, chemical taxonomy/ontology, biophysical properties, references, external links and many other data components needed to fully describe a metabolite or its provenance. Every metabolite in MiMeDB has data entered into these major data fields, which are often subdivided into 5–10 other data categories.

**Figure 1.**
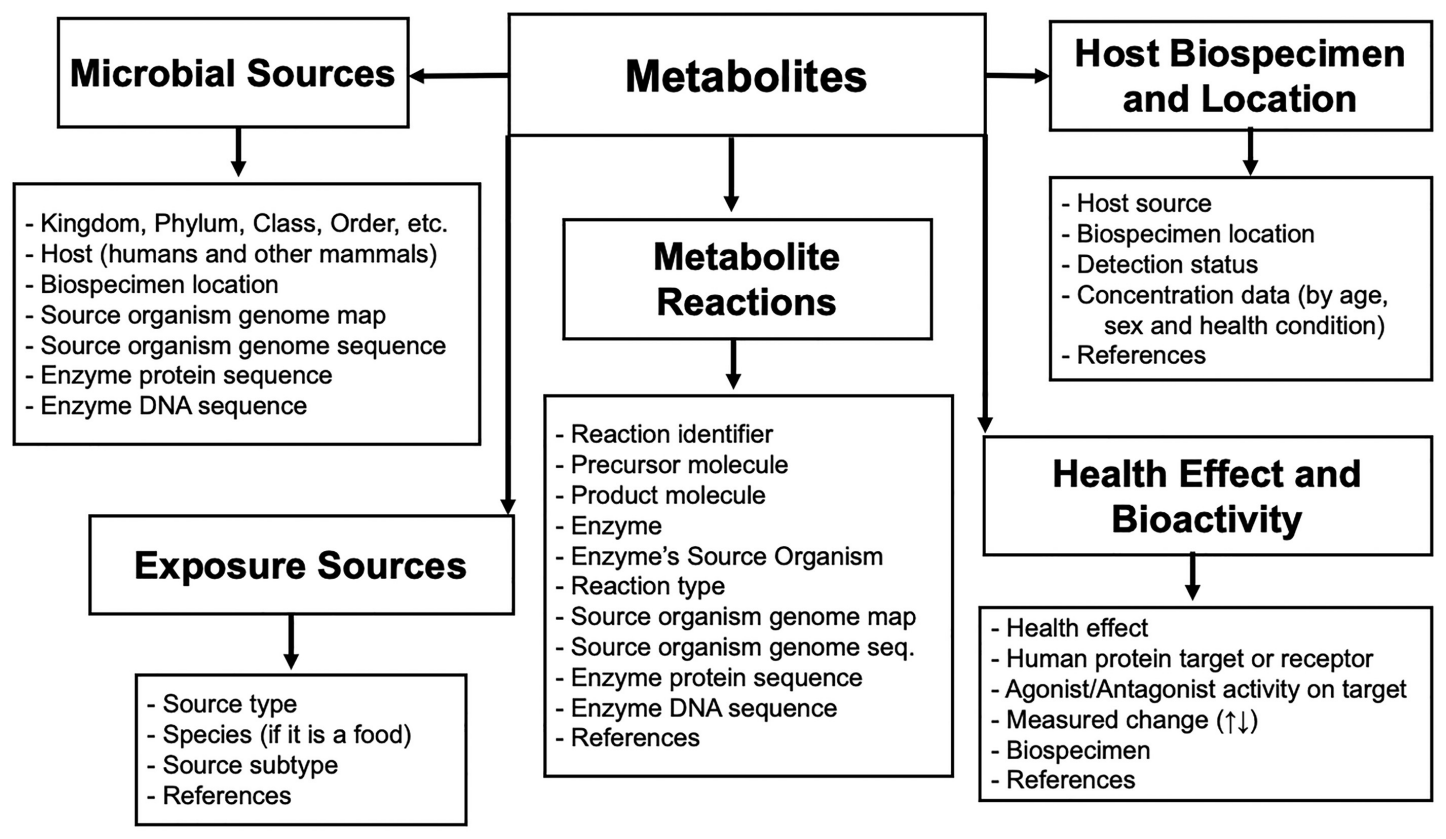
Relationship diagram of the backend structure of the Human Microbial Metabolome Database (MiMeDB).

The other five data categories in MiMeDB also contain their own set of major data fields. The ‘Microbial Sources’ category contains data fields for each microbe's Superkingdom, Kingdom, Phylum, Genus/Species, Genome map, and Host(s). The ‘Biospecimens & Location’ category contains data fields for each metabolite's Host source, Biospecimen location, Detection status (detected and quantified, detected only), Concentration data (for a given age, sex and health condition) and the associated References. The ‘Exposure Sources’ category contains data fields for external sources or exposures for a given compound indicating the Source type, the Species (if it is a plant or animal-derived food), the Source subtype and the relevant References. The ‘Health Effects’ category contains data fields for the metabolite's Health effect, the Human protein target or receptor, the Agonist/Antagonist activity on the target, the Measured change (increased or decreased), the Biospecimen and the associated References. The ‘Metabolic Reactions’ category contains data fields for the metabolite's Reaction identifier, the Precursor molecule, the Product molecule, the Enzyme, the Enzyme's source organism, the Reaction type, and the relevant References. A diagram illustrating the relational structure of MiMeDB’s backend data is provided in Figure 1.

## MiMeDB INTERFACE AND FRONTEND DESIGN

**Figure 2.**
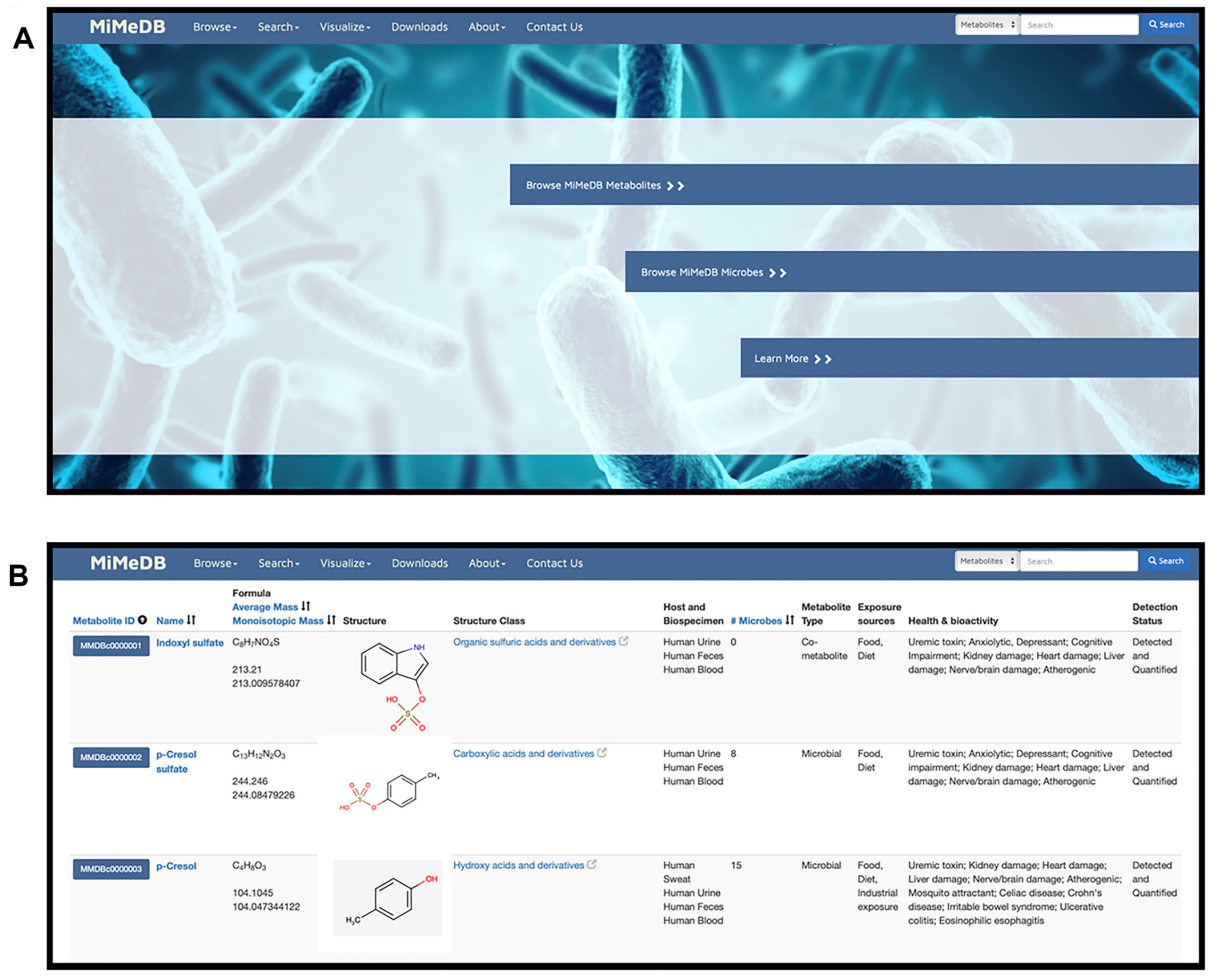
Screenshots from the Human Microbial Metabolome Database (MiMeDB) showing the (**A**) MiMeDB Homepage and (**B**) the MiMeDB Metabolite Table.

MiMeDB has been designed using many of the same interface features and a very similar layout as many of our more popular metabolomic databases such as the HMDB (23), ECMDB (24) and the Yeast Metabolome Database (YMDB) (27). A screenshot of the MiMeDB homepage is shown in Figure 2A. As seen in this image the website's navigation bar (located at the top) consists of six menu options: **Browse**, **Search**, **Visualize**, **Downloads**, **About** and **Contact Us**. On the right side is a general text search box where users may enter text to search the entire MiMeDB database (explained further below). Below the navigation bar is a set of three coloured hyperlink bars that allow users to instantly access the most popular browsing tools in MiMeDB, namely: (i) *Browse MiMeDB Metabolites*, (ii) *Browse MiMeDB Microbes* and (iii) *Learn More*. Clicking these hyperlink bars will take users to the Metabolite Browser, the Microbe Browser or the About page on MiMeDB, which provides a brief tutorial on how to use the database.

MiMeDB offers a wide range of database browsing options. These are listed under the Browse menu tab and include options to browse by: (i) Metabolites; (ii) Microbes; (iii) Biospecimens & Locations; (iv) Health Effects; (v) Exposure Sources and (vi) Metabolic Reactions. Selecting ‘Metabolites’ will produce a sortable table, called the ‘Metabolite Table’. This table lists all the metabolites in MiMeDB with several data filtering options provided in a menu located above the table. Using the filter menu, users may choose to filter the displayed ‘Metabolite Table’ by Host, Biospecimen, Metabolite Type, Exposure Sources, Health Effects, Structure Class and Detection Status. Each filter option provides a pulldown menu with which users may choose among several named options. The default for all filters is ‘All’, which means the Metabolite Table will display all options. In other words, no filter is applied. Users may select any combination of the filtering options. After choosing the filter options, users must click the blue ‘Apply Filter’ button on the lower right side of the filter menu. This will generate a new Metabolite Table with the appropriate filters applied. A new Metabolite Table can be generated by pressing the dark grey ‘Clear’ button on the filter menu and selecting a different set of filter options.

The Metabolite Table displays 11 columns (Figure 2B) describing each metabolite or chemical in MiMeDB. These include the Metabolite ID (or MiMeDB compound identifier), the Name, the Molecular Formula, the Mass (Average and Isotopic), a thumbnail image of the Structure, the Structure Class, the Host and Biospecimen, the Number of Microbes, the Metabolite Type, the Exposure Sources, the Health Effects and the Detection Status. The Structure Class refers to the chemical class to which the compound belongs using the ClassyFire taxonomy (28). The Host and Biospecimen identifies in which mammal(s) the metabolite has been formally identified and in which type of biological specimen it has been detected. The Metabolite Type refers to whether the metabolite is purely microbial, exogenous or a host-microbe cometabolite, the Exposure sources refers to the probable external sources of the compound, the Health Effects refers to the diseases or conditions that are associated with abnormal concentrations of that metabolite and the Detection status refers to whether the metabolite has been detected only or detected and quantified. Both the dark blue MiMeDB ID button and the dark blue metabolite names are hyperlinked to the MetaboCard for that compound. Users can sort the Metabolite Table according to the Name (alphabetical), Mass (average or isotopic), Compound Class (alphabetical) and the Number of Microbes in which the given metabolite is produced. A metabolite with ‘0’ for the Number of Microbes is a compound that is either exogenous (food, drug or cosmetic) or a host-microbe cometabolite.

**Figure 3.**
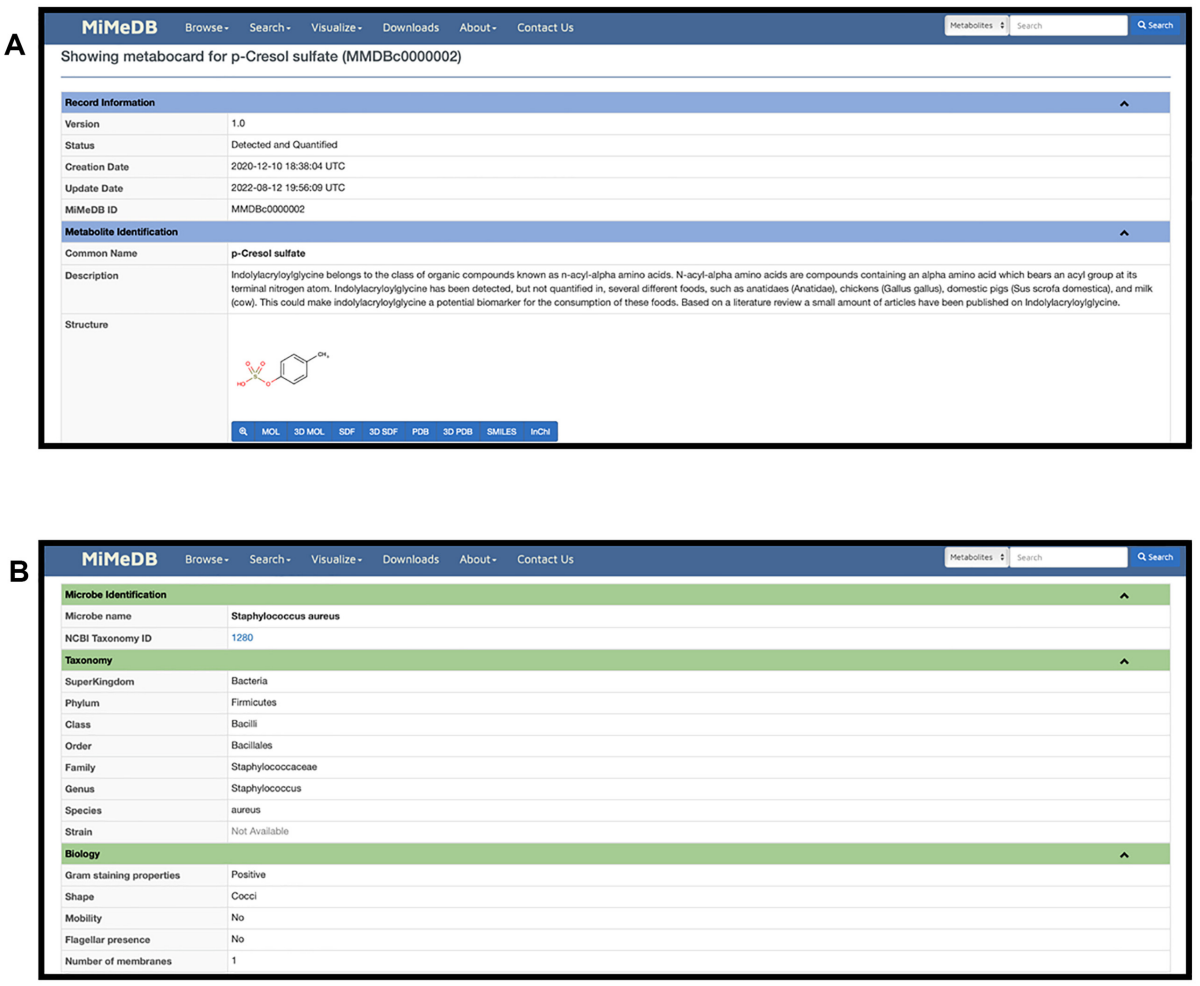
Screenshots showing the (**A**) MiMeDB MetaboCard for p-Cresol sulfate (MMDBc0000002) and (**B**) the MiMeDB MicrobeCard for *Staphylococcus aureus* (MMDBm0000423).

If users click on the MiMeDB compound button or the compound name, they will be taken to the MiMeDB MetaboCard for that compound or metabolite (Figure 3A). Each MiMeDB MetaboCard contains 16 data fields. These include 11 compound-specific data fields: (i) MiMeDB Record Information; (ii) Metabolite Identification; (iii) Chemical Taxonomy; (iv) Functional Ontology; (v) Physical Properties; (vi) Spectra; (vii) Biological Properties; (viii) Human Proteins and Enzymes; (ix) Human Pathways; (x) External Links and (xi) References. In the displayed MetaboCard, these compound-specific data fields are colored in dark blue with some of them (Metabolite Identification and Record Identification) always opened by default. Another five microbe/health effect fields marked with different coloured bars are also displayed including: (xii) Health Effects; (xiii) Biospecimens & Locations; (xiv) Microbial Sources; (xv) Exposure Sources; and (xvi) Metabolic Reactions. Each of these data fields may be expanded or contracted by clicking the down arrow on the right side of each of the coloured bars with the corresponding data field name. Details on the content and table structures within each of these data fields is provided under the MiMeDB **About** tab and the MiMeDB tutorial.

As seen under the **Browse** tab, users may also browse MiMeDB by Microbes. Selecting this option produces a sortable ‘Microbe Table’, similar in layout and design to the Metabolite Table described earlier, containing all microbes in MiMeDB. The Microbe Table also offers several data filtering options provided in a menu located above the table. Using the filter menu, users may choose to filter the displayed Microbe Table by Host, Biospecimen, Microbe Pathogenicity, Superkingdom, Phylum, Oxygen Preference, Energy Production, Gram Stain, Cell Shape and Cell Arrangement. Each filter option provides a pulldown menu with which users may choose among several named options. Filtering in the Microbe Browser works the same as the metabolite filtering in the Metabolite Browser. The Microbe Table displays nine columns describing each microbe in MiMeDB. These include the Microbe ID (or MiMeDB metabolite identifier), the Microbe Name, the Superkingdom, the Kingdom, the Phylum, the Oxygen Preference, the Energy Production, the Host & Biospecimen and the Pathogenicity status. Users can sort the Microbe Table according to the Microbe Name (alphabetical), Superkingdom/Kingdom/Phylum (alphabetical), Oxygen Preference (alphabetical) and Energy Production (alphabetical).

If users click on any MiMeDB microbe button or the Microbe name, they will be taken to the MiMeDB MicrobeCard for that organism (Figure 3B). Each MiMeDB MicrobeCard contains nine data fields including: (i) MiMeDB Microbe Identification; (ii) Microbe Taxonomy; (iii) Microbe Properties; (iv) Host & Biospecimens; (v) Health Effects; (vi) Related Metabolites; (vii) Metabolic Reactions; (viii) Genome Data and (ix) Source Links. Each of these data fields may be expanded or contracted by clicking the down arrow on the right side of each of the coloured bars with the corresponding data field name. Additional details on the content and table structures within each of these data fields is provided under the MiMeDB **About** tab and the MiMeDB tutorial.

Similar browsing options, similar layouts and similar data tables are also available for each of the other **Browse** menu options including ‘Biospecimens & Locations’, ‘Health Effects’, ‘Exposure Sources’ and ‘Metabolic Reactions’. Each browser has appropriate MiMeDB identifiers, sortable tables and field-expandable viewing cards (named ReactCard, BiospeCard, HealthCard and ExposoCard). Details on the content and table structures within each of these Browsers and their corresponding data fields is provided under the MiMeDB **About** tab and the MiMeDB tutorial.

In addition to the many browsing and data filtering options already described, MiMeDB also provides a number of **Search** utilities. On the upper right-hand corner of the main page, a text search box is available that allows users to search MiMeDB either for Metabolites by name (the default) or Microbes by name. A pull-down menu allows users to select between the two search options. After typing the desired search term in the box, users must press the blue ‘Search’ button to activate the text search. As with the other text search utilities, an auto-suggest feature is provided to help facilitate the search and perform spelling corrections. A question mark on the left of the pull-down menu provides advice for users for performing smarter or more efficient text searches. MiMeDB offers many other search options located on the top navigation bar via the **Search** menu tab. These options include a ChemQuery Structure Search (for chemical structure searches), a Molecular Weight Search, a standard Text Query Search, an Advanced Search (supporting advanced text searches covering >20 different data field categories), a wide variety of LC-MS, GC-MS and NMR Searches, as well as DNA and Protein Sequence Searches. The spectral and structure searches are identical to those described for HMDB (23). The sequence search uses BLASTN for DNA sequence searches and BLASTP for protein sequence searches (29). All returned sequence matches are hyperlinked to the relevant genome map view of the top scoring microbes. The ChemQuery Structure Search utility uses the MarvinView Applet from ChemAxon, which allows users to interactively draw structures (or paste in SMILES strings) into an interactive drawing pallet and to search for similar chemical structures using the Tanimoto similarity index.

**Figure 4.**
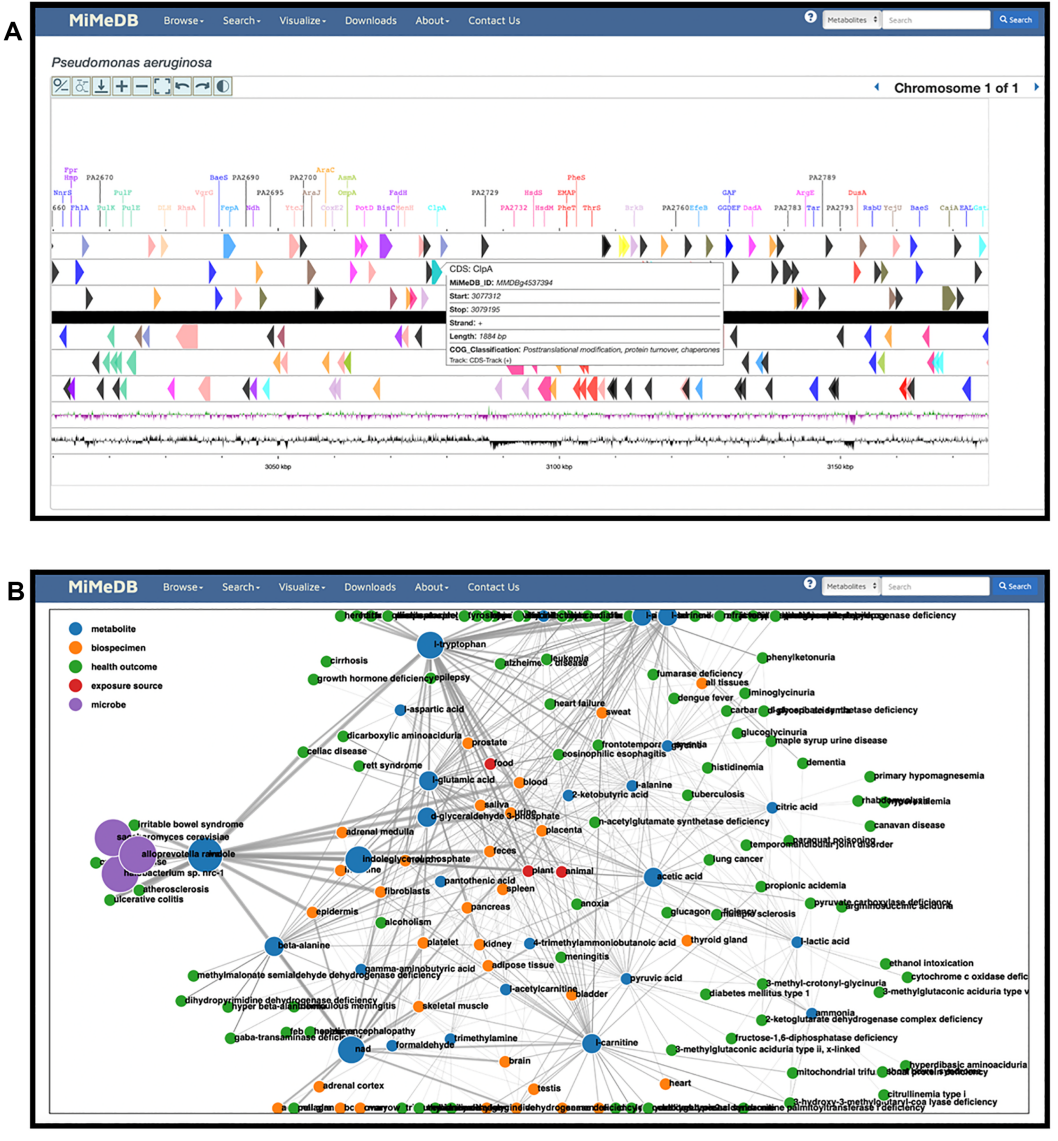
Screenshots showing the (**A**) MiMeDB Chromosome Viewer for a portion of the *Pseudomonas aeruginosa* genome and (**B**) the MiMeDB Network Viewer highlighting connections with L-tryptophan metabolism.

Also located in MiMeDB’s upper navigation bar is the **Visualize** tab, which offers two data visualization options: (i) Chromosome Viewer and (ii) Network Viewer. Chromosome Viewer allows users to interactively view individual microbial chromosomes (bacterial and eukaryotic) using zoomable mapping tool based on CGView (30). CGView is a widely used JavaScript tool that allows the visualization of both circular and linear chromosomes along with their genome/proteome annotations. To access a given microbial genome in MiMeDB, users must enter the microbe's name (genus + species) or partial name (which uses an auto-suggest function to complete the name) or a GenBank Identifier (GI) to select the microbial genome or chromosome to view. Once entered, users must press the blue ‘View’ button to generate the chromosomal map. MiMeDB’s Chromosome Viewer displays both the forward and reverse strands of each chromosome(s) along with the position, name and orientation of each annotated gene (if users zoom in sufficiently). Zooming, recentering and chromosome rotation functions are provided as clickable widgets on the upper corner of the viewer. All genes are colored using the Clusters of Orthologous Genes (COG) functional categories which are displayed as a legend beside the displayed chromosome. Through the ‘Search’ box located underneath the chromosome map, users can either search for a gene/protein by name or by sequence by selecting from the pull-down menu adjacent to the Search box. If searching by name, users must enter a gene abbreviation or protein name (supplemented by an auto-suggest function) or a COG functional category. If searching by sequence, users must enter a DNA or protein sequence within the text box and press ‘BLAST’. Either query will cause the viewer to display or highlight the entered or matching gene on the chromosome map. Clicking on the highlighted gene name generates a pop-up GeneCard that provides detailed information about the selected gene's exact start and end position, its DNA sequence, its protein sequence (if it is a translated gene), its protein name, the gene length, the protein length, the protein molecular weight, the gene function(s) (via COG) and other metadata about the gene and/or protein. A screenshot of MiMeDB’s Chromosome Viewer is shown in Figure 4A.

MiMeDB’s Network Viewer allows users to interactively construct and view networks connecting metabolites to health effects to exposures to microbes to body sites, etc. To use the Network Viewer, users must enter a few details on the Network Specification box. Under the ‘Select Microbe’ box, users must enter one or more microbe names (genus + species) or partial names (using the auto-suggest function) or GenBank GI numbers. To limit the load on the server, a maximum of five microbes can be entered. Under the ‘Select Metabolite’ box, users must a chemical or metabolite name (again typing in a name or partial name or the MiMeDB compound identifier). The selected metabolite serves as the hub upon which the network diagram is centred. Users may also select the ‘Degree of Connectivity’ (default of 5) from the pull-down menu. After pressing the ‘View’ button an interactive, zoomable network map is generated. This is created using a JavaScript network viewer developed in-house using JavaScript and the D3 JavaScript library. The displayed network diagram builds out from the known metabolites, exposures and health effects within MiMeDB that have links to the entered metabolite and to the entered microbe(s) and the network constraints. The number of connections or links between the entered microbe and metabolite nodes defines how large or complex the network diagram can be. A color legend is provided that allows different nodes (metabolites, microbes, exposures, body sites, etc.) to be easily distinguished. To limit the size of certain exposure categories (esp. food), Network Viewer will consolidate some items into obviously named categories. A screenshot of MiMeDB’s Network Viewer is shown in Figure 4B.

MiMeDB also has a **Download** tab, an **About** tab and a **Contact Us** tab. The **Download** tab allows users to download most of the MiMeDB data including metabolite structures, chemical metadata, spectral data, DNA and protein sequences, reactions, microbe lists and microbe taxonomy. Each downloadable file provides information on the file type and data size. The **About** tab has a submenu that provides an overview on MiMeDB, database statistics, citation information, a brief tutorial on how to use MiMeDB, data sources, tables on MiMeDB data structures and information on its FAIR compliance.

## MiMeDB CODING, ASSEMBLY, CURATION, QUALITY CONTROL AND FAIRNESS

The MiMeDB website was designed using the features found in most modern web-based systems. It uses standardized frameworks and caching systems to make the website more user friendly and responsive. Like other databases developed by our group, MiMeDB uses Redis-based caching that makes the loading of data, structures, images and sequences very fast. To facilitate rapid prototyping and development, the entire MiMeDB database was built upon an MVC (Model-View-Controller) framework called Ruby on Rails (version 6.0.3). In this MVC framework, models respond and interact with the data by connecting to the database, views create the interface to show and interact with the data, and controllers connect the user to the views. Such a framework allowed our database developers to easily create code for much of MiMeDB. This framework is particularly robust and code can be reused in different functions or changed easily to accommodate future plans or abrupt changes in design. In particular, this allowed our development team to liberally borrow code and functions from other databases developed in our lab (23,24,27).

The data in MiMeDB was assembled using a combination of manual annotation, computer-aided literature mining, automated genome annotation, and computer-aided data harvesting of specialized, open-access databases. A complete listing of all major data sources is provided on the top navigation bar via the **About** menu tab under the ‘Data Sources’ section. We should note that many of the specialized open-access databases that were ‘harvested’ were originally built by our own team. MiMeDB was assembled with the same quality assurance, quality control and quality management procedures implemented for all the databases developed by our group, including HMDB (23), DrugBank (31), YMDB (27) and ECMDB (24). This includes careful training of the curators, careful tracking of the provenance (references, database identifiers, sources) of all data, frequent reviews of data quality and completeness by supervisory curators and secondary checks of the data by independent database reviewers. To ensure both completeness and correctness, each metabolite record entered into MiMeDB was reviewed and validated by one member of the curation team after being annotated by a second member. Other members of the curation group routinely performed additional spot checks on each entry.

A number of locally developed software packages including text-mining tools, physico-chemical parameter calculators, spectral predictors as well as chemical, gene and protein annotation tools (DataWrangler, ChemoSummarizer, BioSummarizer) were used to facilitate data entry and data validation (23). To monitor the data entry process, all of MiMeDB’s data is entered into a centralized, password-controlled database. This ensures that all changes and edits to MiMeDB are monitored, time-stamped and automatically transferred. Most senior members of the MiMeDB curation team were PhD-level scientists while junior curators were required to have at least three years of undergraduate training in bioinformatics or molecular biology. This ensured that junior annotators/curators had sufficient biological and/or biochemical knowledge to understand the scientific literature and related data to be entered into MiMeDB. Curation team members met weekly throughout MiMeDB’s two-year development period to design the database architecture, coordinate database development, track team member activities, perform quality assurance checks and ensure that tasks were completed in a timely manner.

Improvements and updates to MiMeDB’s content are done on a continuous basis. Minor corrections or small additions to a MiMeDB entry or its layout are typically done without a formal update announcement. However, all changes are tracked internally and external users can see from the last update date when any changes occurred. As this is only version 1.0 of MiMeDB, all MiMeDB entries are dated with August 2022 as the last update date. Large-scale updates and improvements to the database in the future will be given database version numbers (2.0, 3.0, etc.) and suitable database update dates.

MiMeDB is FAIR compliant (32) and details regarding its ‘FAIRness’ are provided under the ‘About’ menu tab. To ensure findability, all metabolite, microbe, reaction and health effect entries in MiMeDB have a unique and permanent seven-digit MMDB identifier along with a single letter code to distinguish the type of entry. To ensure accessibility, MiMeDB not only provides a well-supported web-based user-interface with extensive search functions, it also provides an application programming interface (API) located under the ‘About MiMeDB’ menu tab, to support programmatic access to the data. To ensure interoperability, all diseases or conditions in MiMeDB are mapped to established ontologies (Disease Ontology, SNOMED CT and ICD-10 [International Classification of Diseases, version 10]). Furthermore, all microbes are linked to NCBI Taxonomy entries and GenBank GI numbers, all sequences are linked to GenBank or UniProt entries and all molecular or microbial physiological data have clear references to other established reference, meta-data or data resources. An extensive and well-annotated data download section is also provided with most data available in standard comma separated value (CSV), structure data file (SDF) or extensible markup language (XML) formats. To ensure re-usability, all data in MiMeDB is extensively sourced with clear provenance. The data in MiMeDB are released under the Creative Commons (CC) 4.0 License Suite according to the Attribution BY and Non-Commercial NC licensing conditions.

## MiMeDB LIMITATIONS AND FUTURE PLANS

MiMeDB represents one of the most comprehensive, interconnected, multi-omic databases ever constructed. This has made it particularly challenging to design and assemble. As a result, there are some layout styles, features and functions that are either missing or somewhat underdeveloped. No doubt some of these interface and display functions will need to evolve into more refined tools based on both user input and our own development and testing over the coming year. Because both microbiome research and microbial metabolomics are rapidly developing fields, it is also expected that some of the information in MiMeDB will become dated over a relatively short period of time. Certainly, every effort will be made to keep the data in MiMeDB as current as possible. However, given the volume of literature being published in these areas, we anticipate there will be a lag of several months with certain updates. We are also hoping that user feedback will help identify those priority areas needing urgent updating or urgent enrichment.

In terms of the future of MiMeDB, we expect to focus on expanding the number of health effects and exposure sources. These are areas where the data is less complete and still evolving. We also hope to expand the ontological data contained in MiMeDB through more extensive data mining and the use of natural language processing (NLP) of online texts and abstracts. This should also help accelerate the database updating process. We also plan to expand the metabolite library in MiMeDB by using some recently developed computational approaches for predicting metabolic transformations, such as BioTransformer (33) and Metabolic In silico Network Expansions (MINEs) (34). These hypothetical metabolites, along with their corresponding predicted physico-chemical data, will likely be kept in a separate data layer in MiMeDB. Nevertheless, we hope these predicted microbial metabolites may provide useful material for hypothesis testing and microbial metabolite identification.

Overall, we believe MiMeDB represents an important first step in the path to providing the integrated, multi-omic resources needed to advance their understanding of the human microbiome, the human microbial metabolome, the human exposome and their combined impacts on human health and disease.

## DATA AVAILABILITY

MiMeDB is FAIR compliant. An extensive and well-annotated data download section is also provided with most data available in standard *.csv, SDF or XML formats. To ensure re-usability, all data in MiMeDB is extensively sourced with clear provenance. The data in MiMeDB are released under the Creative Commons (CC) 4.0 License Suite according to the Attribution BY and Non-Commercial NC licensing conditions.
